# Intense Inflammation and Nerve Damage in Early Multiple Sclerosis Subsides at Older Age: A Reflection by Cerebrospinal Fluid Biomarkers

**DOI:** 10.1371/journal.pone.0063172

**Published:** 2013-05-07

**Authors:** Mohsen Khademi, Ann M. Dring, Jonathan D. Gilthorpe, Anna Wuolikainen, Faiez Al Nimer, Robert A. Harris, Magnus Andersson, Lou Brundin, Fredrik Piehl, Tomas Olsson, Anders Svenningsson

**Affiliations:** 1 Karolinska Institute, Department of Clinical Neuroscience, Neuroimmunology Unit, Stockholm, Sweden; 2 Umeå University, Department of Pharmacology and Clinical Neuroscience, Umeå, Sweden; 3 Umeå University, Department of Chemistry, Umeå, Sweden; 4 Karolinska University Hospital, Department of Neurology, Stockholm, Sweden; University of Texas at San Antonio, United States of America

## Abstract

Inflammatory mediators have crucial roles in leukocyte recruitment and subsequent central nervous system (CNS) neuroinflammation. The extent of neuronal injury and axonal loss are associated with the degree of CNS inflammation and determine physical disability in multiple sclerosis (MS). The aim of this study was to explore possible associations between a panel of selected cerebrospinal fluid biomarkers and robust clinical and demographic parameters in a large cohort of patients with MS and controls (n = 1066) using data-driven multivariate analysis. Levels of matrix metalloproteinase 9 (MMP9), chemokine (C–X–C motif) ligand 13 (CXCL13), osteopontin (OPN) and neurofilament-light chain (NFL) were measured by ELISA in 548 subjects comprising different MS subtypes (relapsing-remitting, secondary progressive and primary progressive), clinically isolated syndrome and persons with other neurological diseases with or without signs of inflammation/infection. Principal component analyses and orthogonal partial least squares methods were used for unsupervised and supervised interrogation of the data. Models were validated using data from a further 518 subjects in which one or more of the four selected markers were measured. There was a significant association between increased patient age and lower levels of CXCL13, MMP9 and NFL. CXCL13 levels correlated well with MMP9 in the younger age groups, but less so in older patients, and after approximately 54 years of age the levels of CXCL13 and MMP9 were consistently low. CXCL13 and MMP9 levels also correlated well with both NFL and OPN in younger patients. We demonstrate a strong effect of age on both inflammatory and neurodegenerative biomarkers in a large cohort of MS patients. The findings support an early use of adequate immunomodulatory disease modifying drugs, especially in younger patients, and may provide a biological explanation for the relative inefficacy of such treatments in older patients at later disease stages.

## Introduction

Multiple sclerosis (MS) is a chronic inflammatory disease of the central nervous system (CNS) with demyelination and damage to neurons/axons, and is arguably the most common cause of neurological disability in young adults [1], [2], [3]. The disease usually presents as a relapsing-remitting form (RRMS) characterized by transient attacks of neurological deficits with a variable degree of recovery, brain parenchymal inflammation with demyelination and axonal damage in an active plaque(s). Over time the majority of patients with RRMS convert to a secondary progressive disease state (SPMS), and a minority develop a progressive course from onset (PPMS). Both PPMS and SPMS are characterized by progressive development of neurological disability without remission [4].

The extent of axonal injury in MS lesions is associated with the degree of inflammation observed in the CNS [5], [6], [7]. CNS inflammation itself is characterized by resident microglial cell activation and infiltration of blood-derived leukocytes [8]. Inflammatory mediators have crucial roles in leukocyte recruitment and their subsequent inflammatory activation within the CNS parenchyma. A large number of molecules have been implicated in the inflammatory reaction, some of which may serve as biomarkers in different immune-mediated diseases [9]. In MS such biomarkers may be used not only for diagnostic purposes, but also to monitor therapeutic effects [10]. Herein we have selected four different and well-documented cerebrospinal fluid (CSF) biomarkers for concerted evaluation in a large number of CSF samples (Table 1).

**Table 1:** Key features of the selected MS biomarkers.

	Name	Gene ID	Role in MS
**MMP9**	Matrix metalloproteinase 9	4318	Extracellular matrix, collagen and myelin degradation, facilitates leukocyte entry to CNS
**CXCL13**	Chemokine (C–X–C motif) ligand 13	10563	Promotes migration of B lymphocytes, increased expression in MS lesions
**OPN**	Osteopontin (SPP1)	6696	Pleiotropic, pro-inflammatory cytokine, abundantly expressed in MS lesions
**NFL**	Neurofilament-light chain (NEFL)	4747	Released into CSF upon axonal/neuronal damage, levels are elevated in MS following relapse and decrease with effective therapy

Abbreviations: MS, multiple sclerosis; CSF, cerebrospinal fluids; MMP9, matrix metalloproteinase 9; CXCL13, chemokine (C–X–C motif) ligand 13; OPN, osteopontin; NFL, neurofilament-light chain.

The matrix metalloproteinases (MMPs) are a group of zinc-dependent endopeptidases that are important modulators of the extracellular matrix. They are expressed by activated white blood cells and act as inflammatory immune-mediators by facilitating leukocyte entry into the CNS and also contribute to myelin damage by cleavage of extracellular matrix proteins [11]. Elevated levels of MMPs, especially MMP9, are evident in the CSF in a variety of neuroinflammatory diseases, including MS [12], [13], [14]. In a smaller cohort of MS patients with active disease, increased levels of MMP9 were detected by ELISA in approximately half of the patients, and these levels decreased upon treatment with the immunomodulatory drug natalizumab [15], a monoclonal antibody which mediate suppression of leukocyte migration into the CNS.

A second group of important inflammatory immune-mediators are chemokines. Leukocyte recruitment is tightly regulated and involves sequential interactions between adhesion molecules, chemokines and chemokine receptors [16]. B lymphocytes in CSF from MS patients and control subjects with non-inflammatory neurological conditions express chemokine receptors, including CXCR5 [17]. The CXCR5 ligand CXCL13 is present in active MS lesions and its concentration is increased in CSF from MS patients [18], [19], [20]. Increased CSF levels of CXCL13 correlate with disease exacerbations and unfavourable prognosis in RRMS, whilst high levels predict conversion from a clinically isolated syndrome (CIS) to definitive MS [21], [22]. CSF levels of CXCL13 are also decreased following treatment with high-dose methylprednisolone and natalizumab [23].

A third group of immune-mediators involved in the inflammatory cascade occurring in MS comprise cytokines and complement factors. Osteopontin (OPN) is a pleiotropic cytokine that is present in most tissues and body fluids where it participates in diverse physiological and pathological processes such as bone mineralisation, malignant transformation, atherosclerosis, inflammation and immunity [24], [25]. This pro-inflammatory cytokine has been suggested to play a central role in the pathogenesis of MS and is considered to be useful as a biomarker. In MS plaques the OPN transcript is abundantly expressed, and anti-OPN immunoreactivity is evident in microvascular endothelial cells, macrophages, astrocytes and microglia within or adjacent to active plaques [26]. OPN levels in CSF from patients with a CIS and RRMS are increased with active disease, correlating with biomarkers of inflammation and tissue damage in the CNS [27]. In a recent study of RRMS we reported increased CSF concentrations of OPN that were normalized upon treatment with natalizumab [15].

Neurofilament-light chain (NFL) forms the backbone to the neurofilament fibre. NFL is released into the CSF following axonal/neuronal damage and reports on MS disease activity. Measurement of NFL in the CSF represents a useful biomarker that can serve as a generic marker for ongoing axonal injury. CSF NFL levels are elevated following clinical relapses and also give prognostic information [28], [29], [30]. Importantly, we recently demonstrated that CSF levels of NFL in MS patients decreased within 6 months of initiating natalizumab therapy, reflecting both the effectiveness of this treatment for MS and the potential value of NFL for monitoring therapeutic efficacy [31].

The clinical course of MS has been suggested to be age-related and the severity of symptoms, as measured by expanded disability status score (EDSS), is positively correlated with age [32], [33]. MS patients with early disease onset exhibit different clinical features compared to those with late onset [34]. Although patients with younger onset age are predicted to take a longer time to reach a progressive phase than those with older onset, their long-term prognosis is generally less favourable [35].

In the present study we used principal component analysis (PCA) and orthogonal partial least squares (OPLS) modelling to analyse possible associations between a set of promising inflammatory biomarkers (MMP9, CXCL13 and OPN), the acute axonal injury marker NFL and robust clinical and demographic measures in a large cohort of patients with MS. This data-driven approach revealed a conspicuous negative association between age and the levels of the biomarkers analysed.

## Materials and Methods

### Subjects

All CSF samples were obtained from an in-house biobank collected during routine neurological diagnostic work-up. A total of 1066 subjects were included in this study. MS patients (n = 471) fulfilling the McDonald criteria [36] comprised RRMS (n = 384), SPMS (n = 57) and PPMS (n = 30). The CIS group contained 169 subjects. Two control groups were included: individuals with other neurological diseases (OND; n = 203) and patients with inflammatory neurological disorders (iOND; n = 223), including subjects with viral/bacterial infections. Sample sets and demographic data for the patients and controls are presented in tables 2–4. Classification and scoring of MS patients were performed according to standard classification systems. SPMS was defined as an initial relapsing-remitting disease course followed by more than 12 months of continuous worsening of neurological function, with or without occasional relapses. At time of sampling, 67 RRMS patients had received immunomodulatory treatment including interferon (IFN)–β1a (n = 32), IFN–β1b (n = 23), glatirameracetate (n = 8), intravenous immunoglobulins (n = 4), and 2 OND patients and 45 iOND patients had received low doses of oral corticosteroids. Routine determination of oligoclonal bands (OCB) and immunoglobulin gamma (IgG) index in CSF and serum were performed as described previously [37]. Inclusion or exclusion of the four IVIG-treated individuals did not affect the outcome of our study concerning treatment influence on measured IgG-index. The ethical review board of the Karolinska Institute and Stockhom approved the study (Diary Numbers: 2003/2-548 and 2009/2107-31-2) and written informed consent was obtained from all patients. A minute number of cases were at an age below 18, the threshold for being regarded as adult in Sweden. In those cases, the next of kin, caretakers, or guardians signed the written informed consent form allowing use of material for research purposes in context with the lumbar puncture that was done for diagnostic purposes.

**Table 2:** Demographic data of the patients with MS, CIS and controls.

Characteristics	RRMS	SPMS	PPMS	CIS	iOND	OND
**No. of Subjects**	n = 389	n = 54	n = 28	n = 169	n = 223	n = 203
**Mean Age (years)** a **(Range)**	34.3(17–73)	54.6(35–81)	51.7(35–67)	35.9(16–65)	49.6(13–83)	41.1(19–82)
**Female/Male (%)**	(71/29)	(61/39)	(50/50)	(74/26)	(74/26)	(72/28)
**Mean Disease Duration (years)** b **(Range)**	12.0(1–52)	27.5(3–58)	11.6(1–31)	6.9(1–24)	NA–	NA–
**Mean EDSS** **(Range)**	2.3(0–8.0)	4.6(2–7.0)	3.6(1.5–6.0)	1.46(0–6.5)	NA–	NA–
**Mean IgG-index** **(Range)**	0.99(0.4–3.3)	0.83(0.41–2.2)	0.895(0.3–1.84)	0.75(0.36–2.81)	0.58(0.33–1.51)	0.52(0.37–0.75)
**OCB (+/−/NA)**	327/51/11	34/12/8	23/2/3	106/48/15	14/74/135	2/90/111
**No. of CSF cell Counts/L (x10^6^) (Range)**	8.4(0–90)	4.4(0–16)	4.7(0–12)	6.7(0–92)	15.3(0–433)	1.9(0–22)

a Age (in years) refers to age at sampling time point.

b Disease duration (in years) refers to the period from disease onset until year 2011.

Abbreviations: RRMS, relapsing-remitting multiple sclerosis; SPMS, secondary progressive MS; PPMS, primary progressive MS; CIS, clinically isolated syndrome; iOND, other neurological diseases with inflammation; OND, other neurological diseases; EDSS, expanded disability status scale; OCB, oligoclonal IgG bands; NA, not applicable (or available); CSF, cerebrospinal fluids.

**Table 3:** Case breakdown of the model building and model testing data sets for all individuals included.

Groups	Inflammatory Condition	Set 1: Model Building(Complete ELISA data)	Set 2: Model Testing(Incomplete ELISA data)	Total	
**MS**	Yes	224	247	471	
**CIS**	Yes	87	82	169	
**iOND**	Yes	125	98	223	
**OND**	No	92	111	203	
	–	**548**	**518**	**1066**	**Total**

Abbreviations: MS, multiple sclerosis; CIS, clinically isolated syndrome; iOND, other neurological diseases with inflammation; OND, other neurological diseases.

**Table 4:** Case breakdown of the model building and model testing data sets within MS cohort included.

Disease Type	Set 3: Model Building(Complete ELISA data)	Set 4: Model Testing(Incomplete ELISA data)	Total	
**PPMS**	12	18	30	
**SPMS**	22	35	57	
**RRMS**	210	174	384	
	**244**	**227**	**471**	**Total**

Abbreviations: PPMS, primary progressive MS; SPMS, secondary progressive MS; RRMS, relapsing-remitting multiple sclerosis.

### ELISA Studies

CSF samples were centrifuged immediately after sampling and stored frozen at −80°C until analysis. CXCL13, MMP9, OPN and NFL levels were measured using commercially available ELISA kits (Quantikine Human CXCL13/BLC/BCA-1, MMP9 (total), Osteopontin: R&D Systems, Abingdon, UK; NFL: Uman diagnostics, Umeå, Sweden) according to the manufacturers’ instructions. Measurements were performed in duplicates using 50 µl undiluted cell-free CSF per well.

### Multivariate Data Analysis

An underlying assumption of classical statistics is that variables are independent of each other. A correlation matrix (Table 5) of the variables in this study highlighted the existence of relationships between some of the variables within the MS cases and therefore a multivariate analysis approach was considered to be more appropriate.

**Table 5:** Correlation matrix for variables included in the modeling.

Variables	CSF-Mono	Age	IgG-Index	OCB	CXCL13	MMP9	OPN	NFL	EDSS
**CSF-Mono**	1	−0.34668	0.44152	0.17620	0.42257	0.56368	0.24932	0.19249	0.03794
**Age**		1	−0.11521	−0.24442	−0.27036	−0.31236	−0.00539	−0.05608	0.27487
**IgG-Index**			1	0.32719	0.43972	0.50328	0.112975	−0.01256	−0.05362
**OCB**				1	0.34644	0.27027	0.05524	0.09732	−0.06126
**CXCL13**					1	0.66558	0.17992	0.26998	0.03631
**MMP9**						1	0.15147	0.222514	0.00305
**OPN**							1	0.30813	0.24127
**NFL**								1	0.03122
**EDSS**									1

Abbreviations: CSF, cerebrospinal fluids. OCB, oligoclonal IgG bands; CXCL13, chemokine (C–X–C motif) ligand 13; MMP9, matrix metalloproteinase 9: OPN, osteopontin; NFL, neurofilament-light chain; EDSS, expanded disability status scale.

We thus used the multivariate statistical methods PCA and OPLS [38], [39] to create models of the large and complex datasets using SIMCA 13.0 (Umetrics AB, Umeå, Sweden). PCA is an unsupervised analysis method useful for reducing the dimensionality of multivariate data by summarizing variation into orthogonally constrained principal components. The principal components describe structures in the original variables based on size of variance, where the first principal component reveals the largest variation in the data. The principal components are visualized in terms of scores (t) and loadings (p). Scores reveal trends, clusters and outliers among the patients and the loadings give information regarding which of the original variables are responsible for the differences in scores i.e. what is different between patients. OPLS is a supervised method that encompasses linear regression between latent variables (linear combinations of correlated original variables) towards a defined Y (response) variable. OPLS handles variation related to the response variable separate from variation unrelated (orthogonal) to the response variable and the orthogonal components are usually plotted at right angles to the component related to your variable of interest. The loadings from PCA and OPLS models created within SIMCA can be used to predict the scores of a second sample set (new X data) which can contain missing values; in this way a model created using one data set can be validated using a second data set.

## Results

### Sample Selection for Model Building and Model Testing

The full dataset of 1066 individuals contained a variety of clinical, laboratory and biochemical information. In order to create reliable models we divided the dataset into 2 subsets of samples: one set for model building and one for model testing. Only objective measurements or clearly defined biological properties were included in all the multivariate models, namely: CXCL13, MMP9, OPN, NFL, mononuclear cell count in CSF, IgG index, presence of OCB and patient age. Sex was included in initial models but no effect was seen and EDSS was used in some experiments to test for any possible influence. The composition of the sample sets for model building and model testing are summarised in tables 3, 4 and S1. Since it was not possible to perform all ELISAs on samples from all cases, due to limited sample availability, Set 1 (model building) contained only cases with a complete set of ELISA results and Set 2 (model testing) contained cases with data from 3 or fewer ELISAs (Table 3). Thus all models were created based on a complete set of ELISA variables (Set 1) and then used to predict the test set of cases (Set 2). The MS patient subgroup was further divided into 2: Set 3 containing those cases with complete ELISA data and Set 4 for cases with data from 3 or fewer ELISAs (Table 4).

### Exploration of Biomarker Variability in the Full Data Set

**Figure 1:**
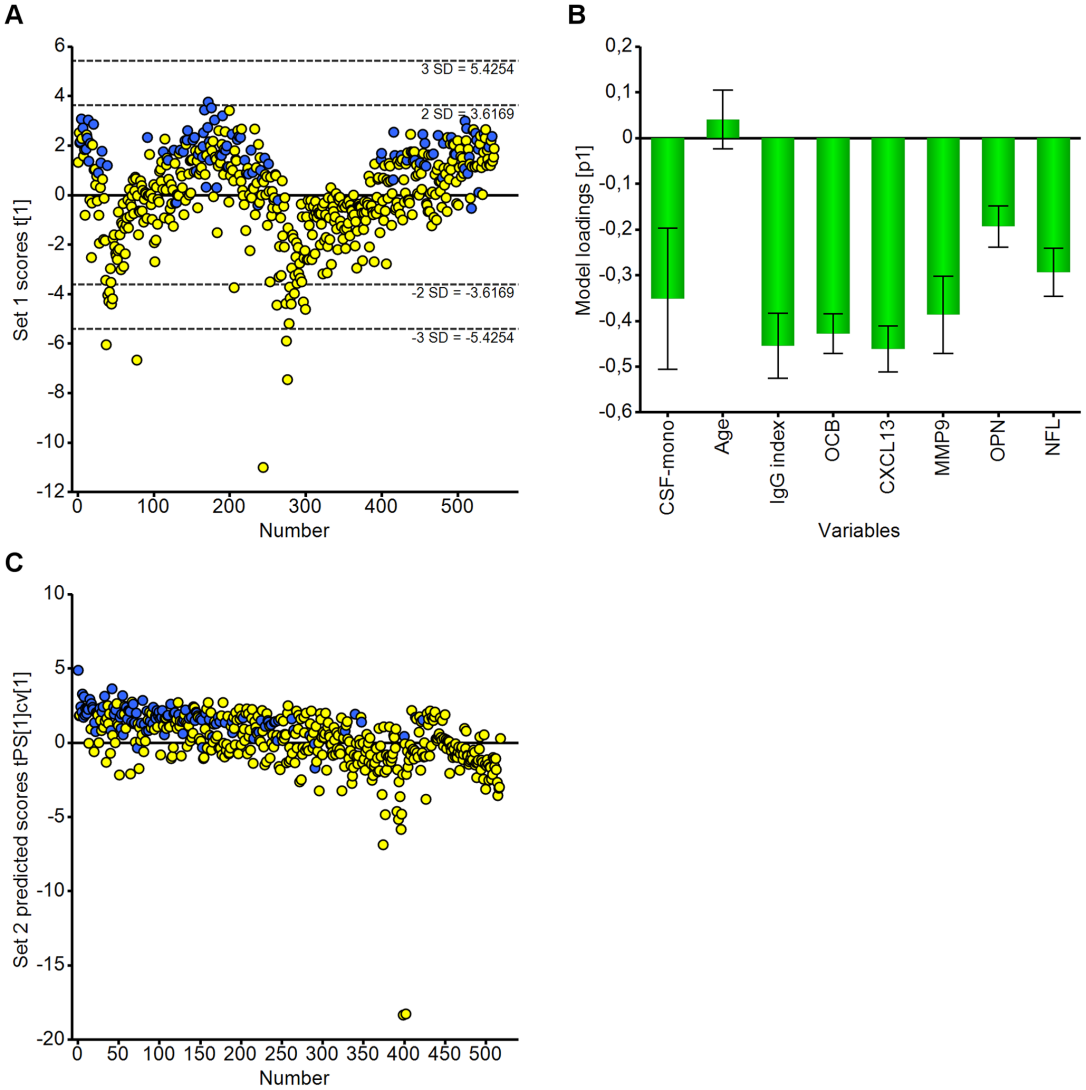
PCA broadly distinguishes between inflammatory and non-inflammatory conditions. PCA scores plot for the model building dataset (Set 1). Most non-inflammatory cases (blue) are associated with positive scores, located in the upper part of the plot. There is more variation in the distribution of the inflammatory cases (yellow) but many associate with negative scores (**A**). The PCA loadings plot shows that all the measured variables except age underlie the observed scores distribution seen in A (**B**). Scores of the test set (Set 2) were predicted using the model derived for Set 1. Non-inflammatory conditions, again, cluster mostly in the upper part of the plot (**C**). Extreme outliers (≥3 SD) in both scores plots belonged to cases of herpes encephalitis and neuroborreliosis.

We explored the variability of the whole data set using PCA on 8 X variables: CSF-mononuclear cell count, Age, IgG index, presence of OCB, CXCL13, MMP9, OPN and NFL (Figure 1B). Models were made using Set 1 data and validated using Set 2 data (Table 3). The resultant model contained one principal component explaining 36.3% of the variation (R^2^ = 0.363, Q^2^ = 0.139). While considerable variation exists between cases, inspection of the scores plot (Figure 1A) reveals a broad localisation of non-inflammatory cases (blue circles) in the high t[1] scores region. Inspection of the corresponding loading plot (Figure 1B) shows that this localisation is due to a negative association with all of the inflammatory and axonal injury markers examined. Extreme outliers (≥3 SD) were cases of herpes encephalitis or neuroborreliosis. Removal of these outliers from the model increased the significance of the age variable there higher age being associated with lower levels of inflammatory/axonal injury markers (data not included). Since the variables included in the model mainly reflect inflammatory activity these results appear both valid and logical. To test the model, we predicted the scores for Set 2 data, the patients without a complete set of ELISA results, (Figure 1C) and determined the same overall pattern as seen in Set 1 (Figure 1A). This demonstrated that despite a high degree of variation in the patient set, this PCA model was able to identify an immunological signature of patients with neuroimmunological disorders based on the expression of inflammatory markers.

### Exploration of MS Cases Reveals an Inverse Association of the Selected Biomarkers with Patient Age

**Figure 2:**
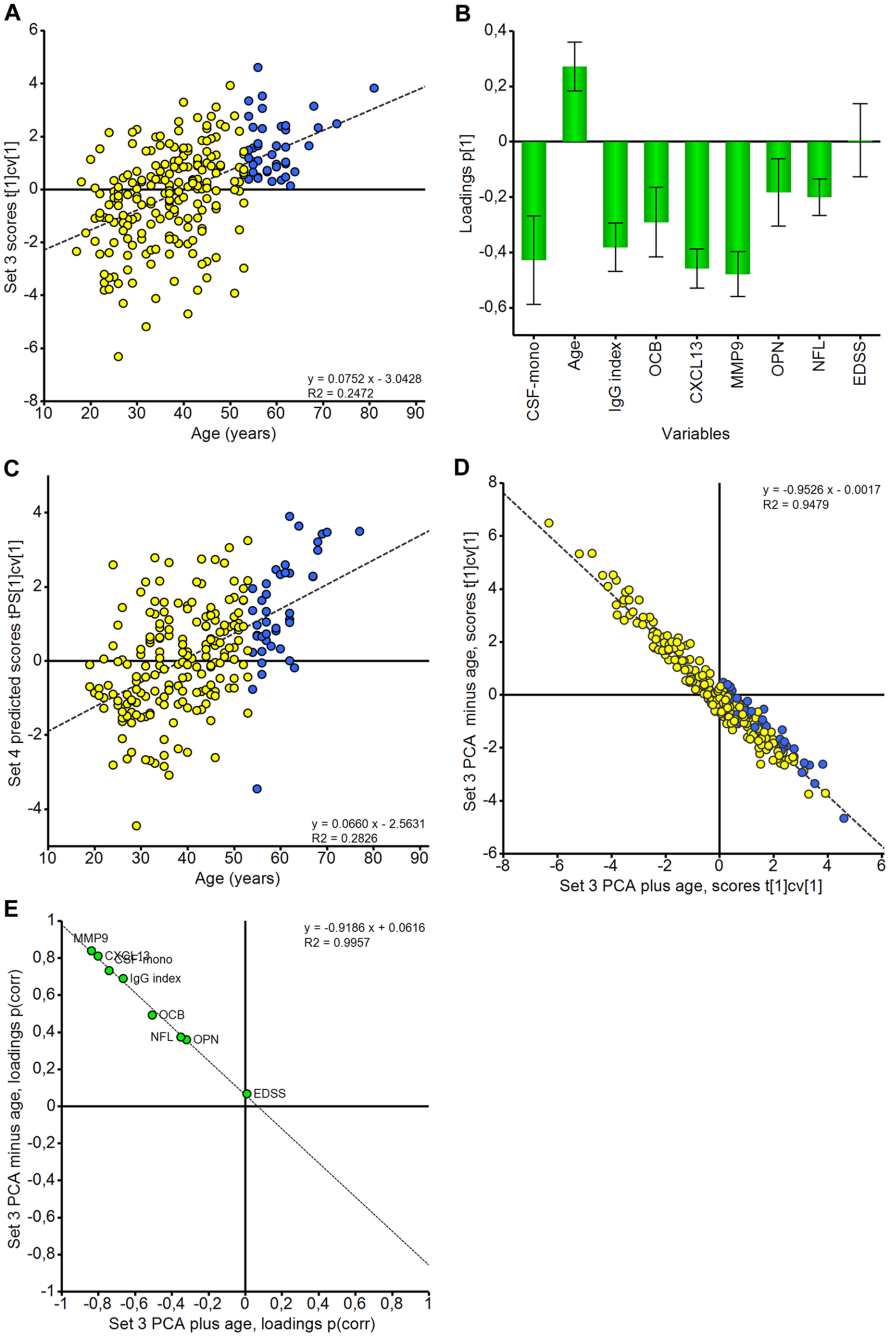
The inflammatory signature for MS is associated with patient age. Scores and loadings respectively for the PCA model of all MS patients with complete ELISA data (Set 3). The loadings show that higher age is associated with lower levels of all inflammatory and axonal injury markers in the CSF. EDSS makes no significant contribution to the model (**A** and **B**). The model derived for Set 3 was used to predict the scores for the test set (Set 4). The scores plot shows a similar distribution to that in 2A (**C**). PCA modelling was repeated for Set 3 samples excluding the age variable (**D** and **E**); SUS-style plots plot the scores (**D**) and loadings (**E**) from the two models against each other. The scores and loadings from the 2 models are highly correlated signifying that the age variable in itself is not artificially driving the scores distribution seen in 2A. Scores are coloured according to age group: ≥54 years (blue circles), ≤53 years (yellow circles). Loadings are coloured green.

**Figure 3:**
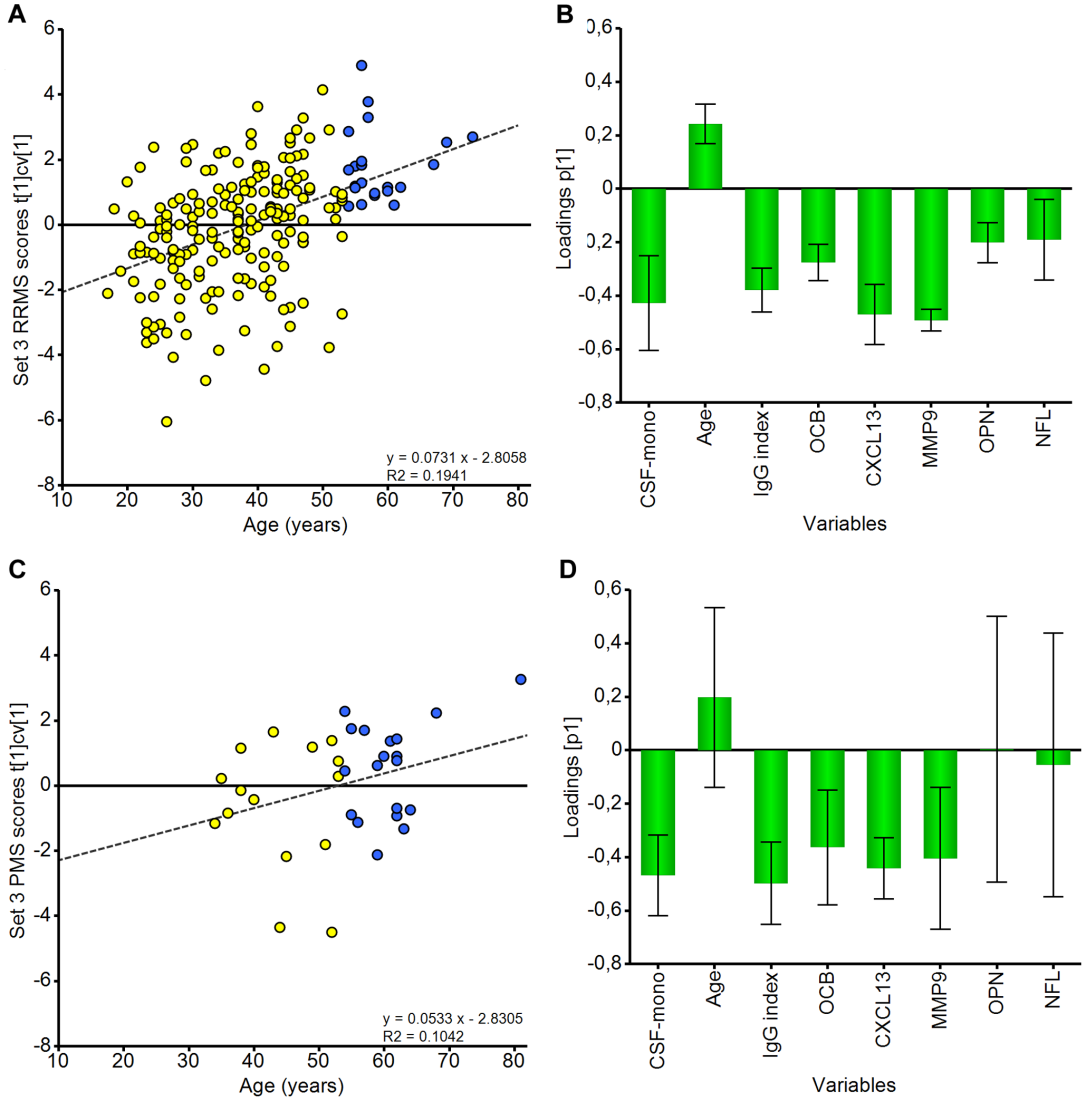
PCA of relapsing-remitting and progressive MS subgroups. Scores and loadings respectively for the PCA model of RRMS patients only with complete ELISA data (Set 3 RRMS patients) (**A** and **B**). Scores and loadings respectively for the PCA model of progressive patients only with complete ELISA data (Set 3 PMS patients) (**C** and **D**). Scores are coloured according to age group: ≥54 years (blue circles), ≤53 years (yellow circles).

**Figure 4:**
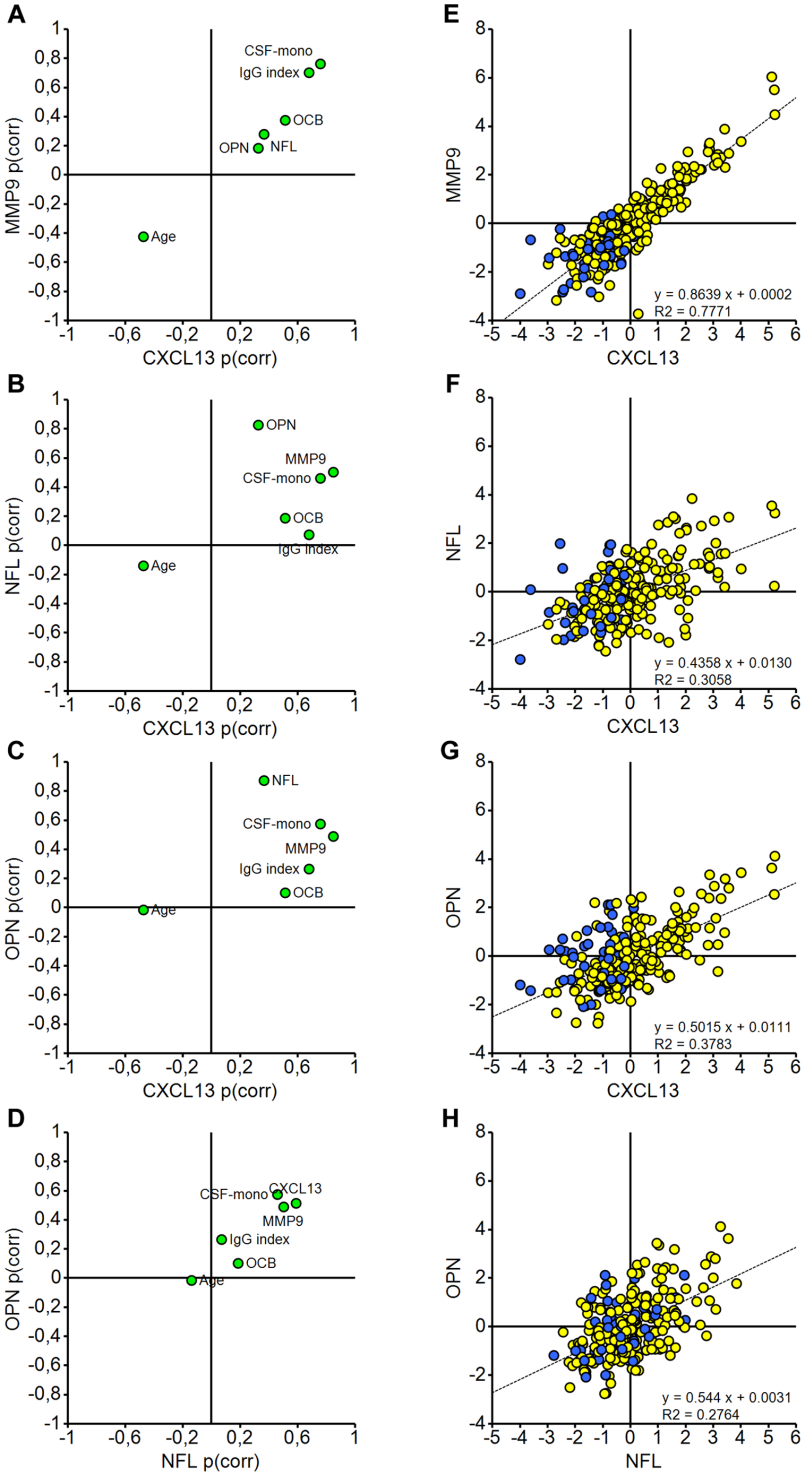
OPLS analysis separates out the variability associated with individual y response variables. SUS plots compare the loadings (p(corr)) from four different OPLS models for all MS patients with complete ELISA data (Set 3) where each of the ELISAs was treated as the y response variable in turn (**A**–**D**). SUS plot methodology was also used to compare the corresponding scores (tcv[1]). The strongest correlation between the scores was seen for MMP9 and CXCL13 (E; R^2^ = 0.7771). Individuals older than 54 years expressed low levels of both markers whereas more variable expression was evident in the younger age group (**E**–**H**). Weaker correlations are seen between the scores for CXCL13 and OPN, CXCL13 and NFL, and NFL and OPN (**F**–**H**). Scores are coloured according to age group: ≥54 years (blue circles), ≤53 years (yellow circles). Loadings are coloured green.

After establishing that PCA could distinguish primary inflammatory from non-inflammatory conditions, we continued the analysis using the MS patient subgroup alone. The cases were separated into two further groups depending on the availability of ELISA data, Set 3 containing complete ELISA data and Set 4 containing data from 3 or fewer ELISAs (Table 4). PCA of Set 3 data using 9 variables (Figure 2B) resulted in a model with 1 significant principal component accounting for 33.6% of the variation (R^2^ = 0.336, Q^2^ = 0.169). When the scores are plotted against patient age it becomes clear that there is an age-dependent distribution, with patients older than 54 years of age having consistently low values of all the analysed CSF markers (Figure 2A). Individuals of 54 years and older were consistently coloured blue in figures 2–4 to enable the reader to follow this group of older patients between the figures. The corresponding loading plot (Figure 2B) shows that CSF inflammatory markers as well as the marker for acute axonal injury, NFL, are negatively associated with age. This indicates that in this model, constructed for MS patients alone, age has a profound effect and acts in an opposite manner to both inflammatory markers and a marker of acute axonal injury. Scores for the model testing set (Set 4) were predicted and exhibited a similar pattern to those for Set 3 (Figure 2C).

A further test of model validity was performed by excluding the key variable of age, repeating the modelling and comparing the results to the previous model. If the new model generated the same pattern in the loading plot this would indicate that the model was not exclusively driven by the excluded variable but rather by the combination of multiple individual variables, thereby increasing confidence in the initial model. The resulting PCA model (R^2^ = 0.359 Q^2^ = 0.175) had a highly similar distribution of scores and loadings to the previous model where age was included. Direct comparison of the two models using shared and unique structures (SUS) plots shows the two models to be highly correlated (Figures 2D,E). This demonstrates that the distributions apparent in the first PCA model are not simply an artefact of including age in the modelling. A latent variable comprising inflammatory/axonal injury variables retains the age distribution of the cases.

Another consistent feature of the models described above is that EDSS does not appear to add any significant contribution to the model in any of the situations tested, i.e. it neither segregates with high values of inflammatory markers/axonal damage or with age (Figure 2B). The most likely explanation for this is that EDSS does not directly reflect ongoing inflammation and nerve injury and is, therefore, of lower value in the interpretation of factors important for disease pathogenesis.

### Exploration of MS Subtypes using PCA

We next examined the MS subtypes in more detail to assess if any associations existed between the measured variables and the clinical phenotypes of RRMS and progressive MS, including both SPMS and PPMS, using PCA (Figure 3). It was clear that RRMS (Figures 3A, B) behaves in an identical fashion to the whole MS population regarding the clustering impact of the analysed parameters, which is perhaps unsurprising since RRMS cases form the majority of patients in the study. In contrast, when analysing progressive MS cases alone (Figures 3C,D), the variables age, NFL and OPN no longer contributed to the model significantly. Thus in this group of progressive patients even younger individuals displayed fewer signs of acute inflammation and axonal injury.

### Examination of Correlations between CSF Biomarkers using OPLS

Finally, we performed a series of supervised analyses to explore correlations between the different CSF markers analysed in this study using the OPLS methodology with SUS plots for visualisation (for OPLS model summaries see Table S2). Classical SUS plots enable the loadings for variation associated with the chosen Y variable of one OPLS model to be plotted against the loadings from another OPLS model. The same methodology was also used to plot the corresponding scores for visualisation and ease of clinical interpretation. This analysis revealed further patterns regarding the distribution of patients aged 54 or older in particular (blue circles). In Figure 4, we present four paired examples of SUS-style scores (tcv) and loadings (p(corr)) plots exploring the correlations between CXCL13 and MMP9 (Figures 4A,E), CXCL13 and NFL (Figures 4B,F), CXCL13 and OPN (Figures 4C,G), and NFL and OPN (Figures 4D,H). The strongest correlation is evident in the scores for CXCL13 and MMP9 (Figure 4E), and highlighting patients aged 54 or over in the same plot, clearly demonstrates that this age group had low values of both markers indicating low inflammatory activity and a lower level of blood-brain barrier damage. Another pattern also emerges of a strong correlation between the levels of CXCL13 and NFL in younger individuals below 54 years of age (Figures 4B,F; yellow circles), while in the older age group CXCL13 levels are consistently low and NFL levels may be either low or high. Therefore, it is likely that high NFL levels, although not as common in older individuals, are not systematically linked to high inflammatory activity and may thus reflect a different pathophysiological mechanism in this age group. The age variable is not essential for explaining variation related to OPN levels (Figures 4C,D), but it is still significantly associated with CXCL13 and to a lesser extent, NFL.

## Discussion

### Markers of Inflammatory Activity and Acute Axonal Injury in a Large Cohort of Patients with MS

The principal finding of the present study is that objective parameters measurable in the CSF display a clear age-dependent pattern in patients with MS and lower values are clearly segregating with older age. This is demonstrated using four different well-documented CSF biomarkers (Table 1) that have been selected based on either their important role as inflammatory immune-mediators by facilitating leukocyte entry into the CNS which in turn contribute to myelin damage or being a marker that reflect acute axonal injury [11], [18], [19], [20], [26], [28], [29], [30]. Despite a large degree of variation, it is of particular importance that the inflammatory activity appears to be highest in those of youngest age and also that this is accompanied by acute axonal injury as measured by CSF levels of NFL. Our findings provide strong support for an early and adequate therapeutic intervention in MS. Since the inflammatory component of RRMS is highly amenable to immunomodulatory treatment, early intervention is necessary in order to minimize axonal damage that in time will become the most important determinant of irreversible functional impairment, brain atrophy and supposed later conversion to a progressive course. Our data are also consistent with previous magnetic resonance studies demonstrating early atrophy or axon loss at early stages of MS [40], [41]. This notion gains further support from our previous work that active immunomodulatory treatment with natalizumab reduces CSF NFL to levels similar to those present in healthy controls [31]. The data from the present study also suggests that clinical measures such as EDSS, at least in the short to medium term, are relatively insensitive instruments with which to monitor disease activity. In this context we also speculate that the higher inherent reserve capacity of younger patients may mask a relatively more severe disease activity compared to older patients. This notion is supported by the observation that the time taken to reach a certain EDSS in general is longer in patients with young onset compared to patients with disease onset as adults [42].

The efficacy of disease-modifying drug treatment is likely to depend on the degree of disease-related inflammation. It has become increasingly clear that currently available treatments for RRMS have limited or no usefulness during progressive disease [43], [44], [45], [46]. Data on the efficacy of more recent additions to the MS disease-modifying drug armamentarium in the later disease stages are still lacking. However, it is of interest to note that an age effect was evident in the randomised controlled trial of rituximab in PPMS, since the rate of progression was lower in the rituximab treatment arm compared to placebo only in patients with age below 51 years (and with signs of neuroradiological activity) [47].

### Age as an Informative Objective Variable along with a Number of Biochemical Measurements in CSF

The understanding of underlying pathophysiological processes in MS is critical for designing the most rational treatment approach to the disease. To achieve this it is essential that appropriate parameters that report on the underlying disease process can be determined as accurately and objectively as possible. Commonly used parameters such as disease onset, disease duration and EDSS do not accurately fulfil these criteria, since the initial point of disease onset is almost impossible to know. Indeed, the initial inclusion of EDSS in the modelling revealed that this measure did not influence the results in any significant direction, indicating that this variable is unrelated to the levels of CSF biomarkers. Age was therefore the only informative objective demographic variable for this study, alongside a number of biochemical measurements from the CSF. A combined panel of CSF biomarkers; IL12p40, CXCL13 and IL–8 has recently been presented as reflecting active intrathecal inflammation, partly based on a PCA approach [20]. These interesting data are in line with our own findings with respect to CXCL13 and a series of previous reports on this chemokine [18], [19], [21]. The data-driven analysis approach used in our current study, combined with a large sample size, has revealed an additional age-dependent aspect of both inflammatory and neuronal/axonal damage markers that was not apparent in the previous studies. This tendency towards higher levels of biomarkers at younger age would be interesting to explore in further studies. Prior investigation into this set of biomarkers with a focus on progressive MS found the levels to be increased compared to cases of non-inflammatory OND [48], which is perhaps at first sight in contrast to the present study. However, the biomarker levels in the progressive cases were always lower than in RRMS, and the progressive patients studied were all under the age cut off (54 years old) used here.

We currently do not know the underlying reason for the decreased intra-CNS inflammation at older age. One attractive possibility could be the phenomenon of immunosenescence. There is a wealth of literature describing decline in T cell function at older ages which may explain the differential inflammatory responses [49], [50], [51].

### Sample Quality/quantity and Treatment Effects

The majority of samples were obtained during diagnostic lumbar puncture, less than 15% of the MS patients being on any type of disease-modifying drug. In this way we were more likely to find patterns linked to the underlying disease process rather than to treatment-induced effects. The large size of the dataset also enabled analyses over a large age range, thereby making it possible to detect the apparent age effect on the studied parameters. The occurrence of increased levels of inflammatory and nerve injury markers is well established in MS [18], [22], [28], [52] but in most datasets it is not possible to perform comparisons between age groups because the division into subgroups will reduce the power of the analyses. With our dataset of more than thousand samples, approximately half being MS patients, it was possible not only to analyse an age effect but also to divide the samples into two datasets in which the second could be used to verify the models.

### Immune Activity Markers as Part of an Age-related Phenomenon

Different age-dependent effects have been previously described in MS pathophysiology [53], [54], [55], [56]. Notably, the occurrence of gadolinium-enhanced lesions have been reported to be associated with young age [53], which is in concordance with our findings since gadolinium enhancement is a well-accepted sign of active inflammation in MS. The conversion to a progressive phase has also been reported to be primarily age-dependent rather than an effect of disease duration [42], [57]. However, to the best of our knowledge this is the first report of immune activity markers in the context of an age-related phenomenon. Furthermore, in parallel with high inflammatory activity, we observed high levels of axonal injury segregating with lower age indicating a higher rate of neurodegeneration during the early stages of the disease rather than in the progressive phase. The relatively low proportion of older patients in the whole data set might represent a potential weak point. Recruitment of greater numbers of older patients to future studies would provide the possibility to strengthen our conclusions. We were not able to combine MRI data with our models since radiological investigations were not performed in a manner from which quantitative measures could be readily extracted. Future studies should aim to incorporate high quality quantitative MRI measurements in order to be able to link the immunological activity to measures of visible pathology. Also during the progressive phase, axonal damage is associated with inflammatory infiltrates [58], and there is also a continued elevation of CSF nitric oxide metabolites [59]. Thus it may well be that biomarkers related to the inflammation in progressive MS are not adequately represented herein.

### Conclusions

In conclusion, accumulating evidence suggests that a patient’s age should be carefully considered when tailoring their treatment for MS, a notion for which our findings provide additional, strong support. Both inflammatory activity and a well characterized marker reflecting the rate of destructive axonal pathology are at their highest among young patients and both these pathological processes diminish considerably around 54 years of age. We argue that young age at onset should prompt a more aggressive treatment approach combined with vigilant follow-up for the expeditious detection of insufficient therapeutic effect. Our study also highlights the importance of early and rapid diagnosis upon the first signs of disease in order to expedite treatment initiation during this phase, where large gains may be achieved from an active treatment approach. Conversely, our data may also support a controlled treatment de-escalation to be considered in older patients since natural diminution of inflammatory activity may indicate that immunomodulatory treatment is no longer effective. Further research is needed to clarify a possible role for CSF measurements of inflammatory and axonal injury markers in the treatment decision process.

## Supporting Information

Table S1 **Further details of the prediction set cases (Set 2 and Set 4): Numbers of cases with individual ELISA data available.**
(DOC)

Table S2 **OPLS model summaries.**
(DOC)

## References

[1] Compston A, Coles A (2002) Multiple sclerosis. Lancet 359: 1221–1231. 11955556 10.1016/S0140-6736(02)08220-X

[2] Compston A, Coles A (2008) Multiple sclerosis. Lancet 372: 1502–1517. 18970977 10.1016/S0140-6736(08)61620-7

[3] Kipp M, van der Valk P, Amor S (2012) Pathology of multiple sclerosis. CNS neurol disord drug targets 11: 506–517. 22583433 10.2174/187152712801661248

[4] Kremenchutzky M, Cottrell D, Rice G, Hader W, Baskerville J, et al (1999) The natural history of multiple sclerosis: a geographically based study. 7. Progressive-relapsing and relapsing-progressive multiple sclerosis: a re-evaluation. Brain 122: 1941–1950. 10506095 10.1093/brain/122.10.1941

[5] Kornek B, Lassmann H (1999) Axonal pathology in multiple sclerosis. A historical note. Brain Pathol 9: 651–656. 10517504 10.1111/j.1750-3639.1999.tb00547.x PMC8098224

[6] Ferguson B, Matyszak MK, Esiri MM, Perry VH (1997) Axonal damage in acute multiple sclerosis lesions. Brain 120: 393–399. 9126051 10.1093/brain/120.3.393

[7] Trapp BD, Nave KA (2008) Multiple sclerosis: an immune or neurodegenerative disorder? Annu Rev Neurosci 31: 247–269. 18558855 10.1146/annurev.neuro.30.051606.094313

[8] Bruck W, Porada P, Poser S, Rieckmann P, Hanefeld F, et al (1995) Monocyte/macrophage differentiation in early multiple sclerosis lesions. Ann Neurol 38: 788–796. 7486871 10.1002/ana.410380514

[9] Tektonidou MG, Ward MM (2011) Validation of new biomarkers in systemic autoimmune diseases. Nat Rev Rheumatol 7: 708–717. 22045310 10.1038/nrrheum.2011.157 PMC3441180

[10] Harris VK, Sadiq SA (2009) Disease biomarkers in multiple sclerosis: potential for use in therapeutic decision making. Mol Diagn Ther 13: 225–244. 19712003 10.1007/BF03256329

[11] Kieseier BC, Seifert T, Giovannoni G, Hartung HP (1999) Matrix metalloproteinases in inflammatory demyelination: targets for treatment. Neurology 53: 20–25. 10408531 10.1212/wnl.53.1.20

[12] Gijbels K, Masure S, Carton H, Opdenakker G (1992) Gelatinase in the cerebrospinal fluid of patients with multiple sclerosis and other inflammatory neurological disorders. J Neuroimmunol 41: 29–34. 1334098 10.1016/0165-5728(92)90192-n

[13] Leppert D, Ford J, Stabler G, Grygar C, Lienert C, et al (1998) Matrix metalloproteinase-9 (gelatinase B) is selectively elevated in CSF during relapses and stable phases of multiple sclerosis. Brain 121: 2327–2334. 9874483 10.1093/brain/121.12.2327

[14] Ram M, Sherer Y, Shoenfeld Y (2006) Matrix metalloproteinase-9 and autoimmune diseases. J Clin Immunol 26: 299–307. 16652230 10.1007/s10875-006-9022-6

[15] Khademi M, Bornsen L, Rafatnia F, Andersson M, Brundin L, et al (2009) The effects of natalizumab on inflammatory mediators in multiple sclerosis: prospects for treatment-sensitive biomarkers. Eur J Neurol 16: 528–536. 19220425 10.1111/j.1468-1331.2009.02532.x

[16] Ransohoff RM, Kivisakk P, Kidd G (2003) Three or more routes for leukocyte migration into the central nervous system. Nat Rev Immunol 3: 569–581. 12876559 10.1038/nri1130

[17] Sorensen TL, Roed H, Sellebjerg F (2002) Chemokine receptor expression on B cells and effect of interferon-beta in multiple sclerosis. J Neuroimmunol 122: 125–131. 11777551 10.1016/s0165-5728(01)00453-2

[18] Krumbholz M, Theil D, Cepok S, Hemmer B, Kivisakk P, et al (2006) Chemokines in multiple sclerosis: CXCL12 and CXCL13 up-regulation is differentially linked to CNS immune cell recruitment. Brain 129: 200–211. 16280350 10.1093/brain/awh680

[19] Kuenz B, Lutterotti A, Ehling R, Gneiss C, Haemmerle M, et al (2008) Cerebrospinal fluid B cells correlate with early brain inflammation in multiple sclerosis. PloS one 3: e2559. 18596942 10.1371/journal.pone.0002559 PMC2438478

[20] Bielekova B, Komori M, Xu Q, Reich DS, Wu T (2012) Cerebrospinal Fluid IL-12p40, CXCL13 and IL-8 as a Combinatorial Biomarker of Active Intrathecal Inflammation. PloS one 7: e48370. 23226202 10.1371/journal.pone.0048370 PMC3511462

[21] Brettschneider J, Czerwoniak A, Senel M, Fang L, Kassubek J, et al (2010) The chemokine CXCL13 is a prognostic marker in clinically isolated syndrome (CIS). PloS one 5: e11986. 20700489 10.1371/journal.pone.0011986 PMC2916843

[22] Khademi M, Kockum I, Andersson ML, Iacobaeus E, Brundin L, et al (2011) Cerebrospinal fluid CXCL13 in multiple sclerosis: a suggestive prognostic marker for the disease course. Mult Scler 17: 335–343. 21135023 10.1177/1352458510389102

[23] Sellebjerg F, Bornsen L, Khademi M, Krakauer M, Olsson T, et al (2009) Increased cerebrospinal fluid concentrations of the chemokine CXCL13 in active MS. Neurology 73: 2003–2010. 19996075 10.1212/WNL.0b013e3181c5b457

[24] Mazzali M, Kipari T, Ophascharoensuk V, Wesson JA, Johnson R, et al (2002) Osteopontin–a molecule for all seasons. QJM 95: 3–13. 11834767 10.1093/qjmed/95.1.3

[25] Wang KX, Denhardt DT (2008) Osteopontin: role in immune regulation and stress responses. Cytokine Growth Factor Rev 19: 333–345. 18952487 10.1016/j.cytogfr.2008.08.001

[26] Chabas D, Baranzini SE, Mitchell D, Bernard CC, Rittling SR, et al (2001) The influence of the proinflammatory cytokine, osteopontin, on autoimmune demyelinating disease. Science 294: 1731–1735. 11721059 10.1126/science.1062960

[27] Bornsen L, Khademi M, Olsson T, Sorensen PS, Sellebjerg F (2011) Osteopontin concentrations are increased in cerebrospinal fluid during attacks of multiple sclerosis. Mult Scler 17: 32–42. 20921238 10.1177/1352458510382247

[28] Lycke JN, Karlsson JE, Andersen O, Rosengren LE (1998) Neurofilament protein in cerebrospinal fluid: a potential marker of activity in multiple sclerosis. J Neurol NeurosurgPsychiatry 64: 402–404. 10.1136/jnnp.64.3.402 PMC2170011 9527161

[29] Norgren N, Sundstrom P, Svenningsson A, Rosengren L, Stigbrand T, et al (2004) Neurofilament and glial fibrillary acidic protein in multiple sclerosis. Neurology 63: 1586–1590. 15534240 10.1212/01.wnl.0000142988.49341.d1

[30] Salzer J, Svenningsson A, Sundstrom P (2010) Neurofilament light as a prognostic marker in multiple sclerosis. Mult Scler 16: 287–292. 20086018 10.1177/1352458509359725

[31] Gunnarsson M, Malmestrom C, Axelsson M, Sundstrom P, Dahle C, et al (2011) Axonal damage in relapsing multiple sclerosis is markedly reduced by natalizumab. Ann Neurol 69: 83–89. 21280078 10.1002/ana.22247

[32] Trojano M, Liguori M, Bosco Zimatore G, Bugarini R, Avolio C, et al (2002) Age-related disability in multiple sclerosis. Ann Neurol 51: 475–480. 11921053 10.1002/ana.10147

[33] Buchanan RJ, Chakravorty BJ, Tyry T, Hatcher W, Vollmer T (2009) Age-related comparisons of people with multiple sclerosis: demographic, disease, and treatment characteristics. NeuroRehabilitation 25: 271–278. 20037220 10.3233/NRE-2009-0525

[34] Deryck O, Ketelaer P, Dubois B (2006) Clinical characteristics and long term prognosis in early onset multiple sclerosis. J Neurol 253: 720–723. 16502213 10.1007/s00415-006-0095-1

[35] Tremlett H, Paty D, Devonshire V (2006) Disability progression in multiple sclerosis is slower than previously reported. Neurology 66: 172–177. 16434648 10.1212/01.wnl.0000194259.90286.fe

[36] McDonald WI, Compston A, Edan G, Goodkin D, Hartung HP, et al (2001) Recommended diagnostic criteria for multiple sclerosis: guidelines from the International Panel on the diagnosis of multiple sclerosis. Ann Neurol 50: 121–127. 11456302 10.1002/ana.1032

[37] Iacobaeus E, Ryschkewitsch C, Gravell M, Khademi M, Wallstrom E, et al (2009) Analysis of cerebrospinal fluid and cerebrospinal fluid cells from patients with multiple sclerosis for detection of JC virus DNA. Mult Scler 15: 28–35. 18805840 10.1177/1352458508096870 PMC2683979

[38] Wold S, Esbensen K, Geladi P (1987) Principal Component Analysis. Chemometrics and Intelligent Laboratory Systems 2: 37–52.

[39] Trygg J, Wold S (2002) Orthogonal projections to latent structures (O-PLS). J Chemometrics 16: 119–128.

[40] De Stefano N, Narayanan S, Francis GS, Arnaoutelis R, Tartaglia MC, et al (2001) Evidence of axonal damage in the early stages of multiple sclerosis and its relevance to disability. Arch Neurol 58: 65–70. 11176938 10.1001/archneur.58.1.65

[41] Ratchford JN, Saidha S, Sotirchos ES, Oh JA, Seigo MA, et al (2013) Active MS is associated with accelerated retinal ganglion cell/inner plexiform layer thinning. Neurology 80: 47–54. 23267030 10.1212/WNL.0b013e31827b1a1c PMC3589201

[42] Confavreux C, Vukusic S (2006) Natural history of multiple sclerosis: a unifying concept. Brain 129: 606–616. 16415308 10.1093/brain/awl007

[43] Meinl E, Krumbholz M, Derfuss T, Junker A, Hohlfeld R (2008) Compartmentalization of inflammation in the CNS: A major mechanism driving progressive multiple sclerosis. J Neurol Sci. 10.1016/j.jns.2008.06.032 18715571

[44] Wolinsky JS, Narayana PA, O’Connor P, Coyle PK, Ford C, et al (2007) Glatiramer acetate in primary progressive multiple sclerosis: results of a multinational, multicenter, double-blind, placebo-controlled trial. Ann Neurol 61: 14–24. 17262850 10.1002/ana.21079

[45] Panitch H, Miller A, Paty D, Weinshenker B (2004) Interferon beta-1b in secondary progressive MS: results from a 3-year controlled study. Neurology 63: 1788–1795. 15557491 10.1212/01.wnl.0000146958.77317.3e

[46] Clinical Trial of Recombinant Interferon-beta-1a in MS (SPECTRIMS) Study Group (2004) Randomized controlled trial of interferon- beta-1a in secondary progressive MS: Clinical results. Neurology 63: 1768–1768.15557486

[47] Hawker K, O’Connor P, Freedman MS, Calabresi PA, Antel J, et al (2009) Rituximab in patients with primary progressive multiple sclerosis: results of a randomized double-blind placebo-controlled multicenter trial. Ann Neurol 66: 460–471. 19847908 10.1002/ana.21867

[48] Romme Christensen J, Bornsen L, Khademi M, Olsson T, Jensen PE, et al.. (2012) CSF inflammation and axonal damage are increased and correlate in progressive multiple sclerosis. Multiple sclerosis (Epub ahead of print). 10.1177/1352458512466929 23178691

[49] Miller RA (1991) Aging and immune function. Int Rev Cytol 124: 187–215. 2001916 10.1016/s0074-7696(08)61527-2

[50] Franceschi C, Bonafe M, Valensin S (2000) Human immunosenescence: the prevailing of innate immunity, the failing of clonotypic immunity, and the filling of immunological space. Vaccine 18: 1717–1720. 10689155 10.1016/s0264-410x(99)00513-7

[51] Linton PJ, Li SP, Zhang Y, Bautista B, Huynh Q, et al (2005) Intrinsic versus environmental influences on T-cell responses in aging. Immunol Rev 205: 207–219. 15882355 10.1111/j.0105-2896.2005.00266.x

[52] Sellebjerg F, Sorensen TL (2003) Chemokines and matrix metalloproteinase-9 in leukocyte recruitment to the central nervous system. Brain Res Bull 61: 347–355. 12909304 10.1016/s0361-9230(03)00097-2

[53] Tortorella C, Bellacosa A, Paolicelli D, Fuiani A, Di Monte E, et al (2005) Age-related gadolinium-enhancement of MRI brain lesions in multiple sclerosis. J Neurol Sci 239: 95–99. 16209877 10.1016/j.jns.2005.08.006

[54] Koch M, Mostert J, Heersema D, De Keyser J (2007) Progression in multiple sclerosis: further evidence of an age dependent process. J Neurol Sci 255: 35–41. 17331540 10.1016/j.jns.2007.01.067

[55] Tremlett H, Zhao Y, Joseph J, Devonshire V (2008) Relapses in multiple sclerosis are age- and time-dependent. J Neurol Neurosurg Psychiatry 79: 1368–1374. 18535026 10.1136/jnnp.2008.145805

[56] Tutuncu M, Tang J, Zeid NA, Kale N, Crusan DJ, et al.. (2012) Onset of progressive phase is an age-dependent clinical milestone in multiple sclerosis. Mult Scle (Epub ahead of print). 10.1177/1352458512451510 PMC4029334 22736750

[57] Confavreux C, Vukusic S (2006) Age at disability milestones in multiple sclerosis. Brain 129: 595–605. 16415309 10.1093/brain/awh714

[58] Frischer JM, Bramow S, Dal-Bianco A, Lucchinetti CF, Rauschka H, et al (2009) The relation between inflammation and neurodegeneration in multiple sclerosis brains. Brain 132: 1175–1189. 19339255 10.1093/brain/awp070 PMC2677799

[59] Danilov AI, Andersson M, Bavand N, Wiklund NP, Olsson T, et al (2003) Nitric oxide metabolite determinations reveal continuous inflammation in multiple sclerosis. J Neuroimmunol 136: 112–118. 12620649 10.1016/s0165-5728(02)00464-2

