# Supplementary material for: Intense Inflammation and Nerve Damage in Early Multiple Sclerosis Subsides at Older Age: A Reflection by Cerebrospinal Fluid Biomarkers
Source: PLoS One. 2013 May 7;8(5):e63172. doi: 10.1371/journal.pone.0063172 (PMC3646751; doi:10.1371/journal.pone.0063172)
Supplement: Table S1 — Further details of the prediction set cases (Set 2 and Set 4): Numbers of cases with individual ELISA data available. (DOC) [file pone.0063172.s001.doc]

**Table S1. Further details of the prediction set cases (Set 2 and Set 4): Numbers of cases with individual ELISA data available.**

| **Groups** | **MMP9** | **CXCL13** | **OPN** | **NFL** |
| --- | --- | --- | --- | --- |
| **MS** | 146 / 227 | 97 / 227 | 146 / 227 | 128 / 227 |
| **CIS** | 66 / 82 | 14 / 82 | 72 / 82 | 73 / 82 |
| **iOND** | 80 / 98 | 13 / 98 | 98 / 98 | 96 / 98 |
| **OND** | 111 / 111 | 10 / 111 | 111 / 111 | 109 / 111 |

Abbreviations: CXCL13, chemokine (C–X–C motif) ligand 13; MMP9, matrix metalloproteinase 9: OPN, osteopontin; NFL, neurofilament-light chain.
