# Supplementary material for: Intense Inflammation and Nerve Damage in Early Multiple Sclerosis Subsides at Older Age: A Reflection by Cerebrospinal Fluid Biomarkers
Source: PLoS One. 2013 May 7;8(5):e63172. doi: 10.1371/journal.pone.0063172 (PMC3646751; doi:10.1371/journal.pone.0063172)
Supplement: Table S2 — OPLS model summaries. (DOC) [file pone.0063172.s002.doc]

**Table S2. OPLS model summaries.**

| **Model Name** | **R2X (cum)** | **R2 (cum)** | **Q2 (cum)** | **Components** | ***p* (CV-ANOVA)** |
| --- | --- | --- | --- | --- | --- |
| **Y = NFL** | 0.547 | 0.175 | 0.141 | 1 + 1 + 0 | 3.01995^e-007^ |
| **Y = CXCL13** | 0.354 | 0.454 | 0.431 | 1 + 0 + 0 | 4.24844^e-030^ |
| **Y = OPN** | 0.562 | 0.132 | 0.0902 | 1 + 1 + 0 | 0.000162445 |
| **Y = MMP9** | 0.454 | 0.560 | 0.529 | 1 + 1 + 0 | 5.85958^e-038^ |

OPLS divides the variability in the data matrix (variability in X) into the systematic variability (R2X) and the residual variability. The systematic variability (R2X) is then further divided into that which is correlated to the chosen Y variable (predictive) and that which is uncorrelated (orthogonal) to Y. The table above records the model summary details for the four OPLS models discussed in the results and in Figure 4. Each model was based on 7 X variables and 1 Y variable. R2 refers to the systematic variability in X or Y (see key below), and Q2 refers to the fraction of the total variation that can be predicted. Higher Q2 values indicate a better predictive capacity, however Q2 values greater than the R2 value indicate that a model has been over-fitted. All Q2 values herein are below the corresponding cumulative R2 values.

Table Key

R2X(cum): Cumulative fraction of X variation modeled using the X model.

R2(cum): Cumulative fraction of Y variation modeled by X in the component, up to the specified component.

Q2(cum): Cumulative Q2 value for the model.

Components: predictive + orthogonal in X + orthogonal in Y.

*p* (CV-ANOVA): Analysis of variance in the cross-validated residuals of the Y-variable; indicates the statistical significance of the model.

Abbreviations: CXCL13, chemokine (C–X–C motif) ligand 13; MMP9, matrix metalloproteinase 9: OPN, osteopontin; NFL, neurofilament-light chain.
